# Five new mitogenomes sequences of Calidridine sandpipers (Aves: Charadriiformes) and comparative mitogenomics of genus *Calidris*

**DOI:** 10.7717/peerj.13268

**Published:** 2022-04-18

**Authors:** Wan Chen, Keer Miao, Junqi Wang, Hao Wang, Wan Sun, Sijia Yuan, Site Luo, Chaochao Hu, Qing Chang

**Affiliations:** 1 School of Life Sciences, Nanjing Normal University, Nanjing, Jiangsu, China; 2 Jiangsu Open University (The City Vocational College of Jiangsu), College of Environment and Ecology, Nanjing, Jiangsu, China; 3 School of Life Science, Xiamen University, Xiamen, Guangdong, China; 4 Nanjing Normal University, Analytical and Testing Center, Nanjing, Jiangsu, China

**Keywords:** Comparative genomics, Phylogenetics, Mitogenome, Genomics, Calidris

## Abstract

**Background:**

The genus *Calidris* (Charadriiformes, Scolopacidae) includes shorebirds known as dunlin, knots, and sanderlings. The relationships between species nested within *Calidris*, including *Eurynorynchus*, *Limicola* and *Aphriza*, are not well-resolved.

**Methods:**

Samples were collected from Xiaoyangkou, Rudong County, Jiangsu Province, China. Mitogenomes were sequenced using the Illumina Novaseq 6000 platform for PE 2 × 150 bp sequencing, and then checked for PCR products. Protein-coding genes were determined using an Open Reading Frame Finder. tRNAscan-SE, MITOS, and ARWEN were used to confirm tRNA and rRNA annotations. Bioinformatic analyses were conducted using DnaSP 5.1 and MEGA X. Phylogenic trees were constructed using maximum likelihood and Bayesian analyses.

**Results:**

We sequenced and annotated the mitogenome of five species and obtained four complete mitogenomes and one nearly complete mitogenome. Circular mitogenomes displayed moderate size variation, with a mean length of 16,747 bp, ranging from 16,642 to 16,791 bp. The mitogenome encoded a control region and a typical set of 37 genes containing two rRNA genes, 13 protein-coding genes, and 22 tRNA genes. There were four start codons, four stop codons, and one incomplete stop codon (T–). The nucleotide composition was consistently AT-biased. The average uncorrected pairwise distances revealed heterogeneity in the evolutionary rate for each gene; the COIII had a slow evolutionary rate, whereas the ATP8 gene had a fast rate. dN/dS analysis indicated that the protein-coding genes were under purifying selection. The genetic distances between species showed that the greatest genetic distance was between *Eurynorhynchus pygmeus* and *Limicola falcinellus* (22.5%), and the shortest was between *E. pygmeus* and *Calidris ruficollis* (12.8%). Phylogenetic trees revealed that *Calidris* is not a monophyletic genus, as species from the genera *Eurynorynchus* and *Limicola* were nested within *Calidris*. The molecular data obtained in this study are valuable for research on the taxonomy, population genetics, and evolution of birds in the genus *Calidris*.

## Introduction

The genus *Calidris* (Charadriiformes, Scolopacidae) currently comprises 23 small to medium-sized species, including shorebirds such as dunlin, knot, long-winged, and relatively short-billed birds (Schoch et al., 2020). Long-distance migratory wading birds form large mixed flocks on coasts (Anderson et al., 2019; Minias et al., 2015). Molecular phylogeny based on mitochondrial and nuclear sequences revealed poorly resolved species relationships within the genus *Calidris*, with shorter Calidridine sandpiper internal branches, indicative of relatively recent rapid radiation (Baker, Pereira & Paton, 2007; Gibson & Baker, 2012). Based on morphology, the spoon-billed sandpiper (*Eurynorhynchus pygmeus*) was classified as the monotypic genus *Eurynorhynchus*. In contrast, molecular studies have suggested that *Calidris* is not a monophyletic genus, as species from *Eurynorynchus* and *Limicola* were nested within *Calidris* (Gibson & Baker, 2012).

The typical mitochondrial genome (mitogenome) of birds is a circular molecule approximately 16 kb in length. It contains 13 protein-coding genes, two ribosomal RNAs (12S rRNA and 16S rRNA), 22 transfer RNAs (tRNAs), and a non-coding control region (Ruokonen & Kvist, 2002). The mitogenome provides a valuable resource for further studies of molecular systematics, population genetics, and comparative or evolutionary genomics because of its features, including small genome size, low sequence recombination, and maternal inheritance (Du et al., 2019; Hu et al., 2020; Li et al., 2016; Pan et al., 2019; Skujina et al., 2016). The evolutionary history of mitogenome rearrangements suggests at least six independent duplication events, followed by partial deletions or loss of one copy in Passeriformes (Caparroz et al., 2018).

Recent advances in next-generation sequencing (NGS) techniques offer new opportunities to rapidly increase the data quality of published bird mitogenomes (Adawaren et al., 2020; Morales et al., 2018; Tamashiro et al., 2019). However, the large number of mitogenomes published routinely has raised questions about their authenticity. Among 1,876 birds, approximately 5.0% of mitogenomes were problematic (Sangster & Luksenburg, 2021). Free access to published DNA sequences revealed that two of the seven mitogenomes published for Charadriidae are not representative of the taxon (Päckert, 2022). Avian mitogenomes have been shown to include nuclear mitochondrial sequences (numt) or lack a large duplication block that was only detected using a long-range polymerase chain reaction (Skujina, McMahon & Hegarty, 2017).

Only a few complete mitogenome from the genus *Calidris* have been released in NCBI (Chen et al., 2019). The lack of available mitogenomes has restricted our understanding of the phylogenetic relationships and evolutionary patterns of *Calidris* species. In this study, we sequenced and annotated the mitogenomes of five species (*Calidris tenuirostris*, *C. alpine*, *C. alba*, *C. subminuta*, and *Limicola falcinellus*). Mitogenomes were sequenced using the Illumina Novaseq 6000 platform, and PCR products of three mitochondrial regions (16S, COI, and control region) were analysed. Comparative analysis of the mitogenomes of other species may provide useful information for understanding evolutionary and taxonomic research on *Calidris*.

## Materials and Methods

### Sample collection and DNA extraction

All procedures described in this study were approved by the Animal Care and Use Committee of Nanjing Normal University (IACUC–20200517). Samples were collected from a derelict and abandoned mist net in Xiaoyangkou, Rudong county, Nantong City, Jiangsu Province, China (32°33′18.74″N, 120°3′0.39″E) in July 2018 (Table 1). After collection, the muscle was initially preserved in 95% ethanol in the field, and then transferred to −20 °C in the laboratory for long-term storage in Nanjing Normal University (Table 1). Total genomic DNA was extracted using a DNeasy Tssue Kit (Qiagen, Germany) following the manufacturer’s instructions.

**Table 1:** Collection information of specimen in this study.

Common name	Species	Specimen number	Length (bp)	Locality
Great Knot	*Calidris tenuirostris*	NJNU-Cten002	16,678	Rudong, Jiangsu (32.5608°N, 121.1692°E)
Dunlin	*Calidris alpina*	NJNU-Calp001	16,791	Rudong, Jiangsu (32.5608°N, 121.1692°E)
Sanderling	*Calidris alba*	NJNU-Calb005	16,642	Rudong, Jiangsu (32.5608°N, 121.1692°E)
Long-toed Stint	*Calidris subminuta*	NJNU-Csub003	16,765	Rudong, Jiangsu (32.5608°N, 121.1692°E)
Broad-billed Sandpiper	*Limicola falcinellus*	NJNU-Lfal001	15,555	Rudong, Jiangsu (32.5608°N, 121.1692°E)

### Library preparation and sequencing

The DNA concentration was determined using a Nanodrop 1000 Spectrophotometer (Thermo Scientific, Waltham, MA, USA). Extracted DNA was sheared to 400–600 bp using an ultrasonic technique and then sent to Novogene (Beijing, China) for sequencing. The sequencing library was produced using the Illumina TruSeq DNA Sample Preparation Kit (Illumina, San Diego, CA, USA) according to the manufacturer’s instructions. The prepared libraries were loaded onto the Illumina Novaseq 6000 platform for PE 2 × 150 bp sequencing at Novogene (Beijing, China).

Before assembly, Illumina raw data were filtered into clean reads, and undesirable reads were removed by fastp v. 0.21 with the following parameters: “-q 15 -u 40 -5 -x -w 40 -f 10 -F 10” (Chen et al., 2018). This filtering step was performed in order to remove duplicated sequences and the reads with adaptors, reads showing a quality score below 20 (Q < 20), and reads containing a percentage of unlabelled base characters (“N”) equal or greater than 10%. *De novo* assemblies of clean reads were conducted in Geneious 10.1.2, using the mitogenome of *Calidris ruficollis* (GenBank number MG736926) as a reference map (Kearse et al., 2012). The aligned contigs (≥80% similarity and query coverage) were ordered according to the reference genome.

To test the accuracy of next-generation sequencing, three regions (16S, COI, and control regions) were amplified using specific PCR primers (2L, 2H; 5L, 5H; 12L, 12H) (Hu et al., 2018). The following specific PCR primers were designed for the control region, based on the sequence-conserved regions, which were identified using multiple alignments of the complete mitogenomes from the genus *Calidris* downloaded from GenBank (Table 2): (L16250: 5’-TTTGCGCCTCTGGTTCCTATG; H511: TGGGGTATCTAATCCCAGTTTG-3’; H79: 5’-ACGGTAAGGTTAGGACTAAGTC-3’). Amplification was conducted using Takara LA Taq (Takara Biomedical, Dalian, China) under the following conditions: 95 °C for 5 min (initial denaturation); followed by 35 cycles of 95 °C for 30 s (denaturation), 50 ± 55 °C for 30 s (annealing), and 72 °C for 1 min (extension); and a final extension at 72 °C for 8 min; and a 4 °C hold (Hu et al., 2017). PCR products were detected by electrophoresis on a 1.0% agarose gel and sequenced with each of the PCR primers by Shanghai MAP Biotech Co., Ltd. (Shanghai, China). The sequences were analysed using ChromasPro software (Technelysium Pty Ltd., Tewantin, Australia). These fragments and next-generation sequencing were assembled into mitogenomes and aligned using DNASTAR software (Madison, WI, USA).

**Table 2:** Composition and skew rate in the forward strand of seven species mitogenome. Newly sequenced mitogenomes in this study are noted with an asterisk (*).

		Proportion of nucleotides (%)						
Species	Accession no.	A	T	G	C	AT content	AT skew	GC skew
*Calidris ruficollis*	MG736926	31.85	24.76	13.48	29.83	56.61	0.13	–0.36
Calidris tenuirostris*	MW160419	30.94	24.80	13.83	30.43	55.74	0.11	–0.38
Calidris alpine*	MW168383	31.20	25.18	13.75	29.81	56.38	0.11	–0.37
Calidris alba*	MW168384	31.45	25.08	13.61	29.86	56.53	0.11	–0.37
Calidris subminuta*	MW168385	31.60	24.43	13.40	30.53	56.03	0.13	–0.39
Limicola falcinellus*	MW160420	31.49	24.80	13.58	30.10	56.30	0.12	–0.38
*Eurynorhynchus pygmeus*	KP742478	31.29	24.85	13.84	30.02	56.14	0.11	–0.37

### Genome annotation and bioinformatics analysis

The tRNA and rRNA genes were identified and annotated using MITOS (Bernt et al., 2013), tRNAscan-SE 1.21 (Lowe & Eddy, 1997), and ARWEN (Laslett & Canbäck, 2008). The reported and annotated mitogenomes of Scolopacidae (Table 2) were used to manually adjust the start and stop positions of 13 protein-coding genes. Initiation and termination codons of protein-coding genes were identified using the Open Reading Frame Finder from the NCBI website using the vertebrate mitochondrial genetic code and then manually corrected.

The value of the nucleotide composition skewness was measured using the following formulas: AT skew = (A – T)/(A + T) and GC skew = (G − C)/(G + C) (Lobry, 1996; Perna & Kocher, 1995). The MEGA X software was used to calculate the number of variable sites, parsimony informative sites, singleton, and average uncorrected pairwise distances for 13 protein-coding genes of seven mitogenomes of *Calidris* (Kumar et al., 2018). Codon usage was estimated using DnaSP 5.1 (Librado & Rozas, 2009). Nucleotide composition analysis was performed using Microsoft Excel 2019. The rates of non-synonymous substitutions (Ka, π modified) and synonymous substitutions (Ks, π modified) for each PCG were determined using DnaSP 5.0 (Librado & Rozas, 2009). The genetic distances between each species based on the 13 protein-coding genes were calculated with MEGA X using the Kimura-2-parameter (K2P) model (Kumar et al., 2018).

### Phylogenetic analysis

Phylogenetic analysis was performed using Bayesian inference (BI) and maximum likelihood analysis (ML). Two species (*Vanellus vanellus* GenBank no. KM577158 and *Tringa glareola*KY128485) were used as outgroups. There were two datasets as follows: (1) concatenated nucleotide sequences of 13 protein-coding genes and 12S and 16S rRNA for nine species, and (2) 12S rRNA, COI, and Cyt *b* of 23 species (Table S1). To determine the optimal partitioning of the data, the best-fit partitioning scheme and the most appropriate nucleotide evolution model for each partition were implemented in PartitionFinder 2 using the greedy algorithm and Akaike information criterion (AICc) (Lanfear et al., 2017). The partitions and models are listed in Table S2. The BI method was performed using MrBayes 3.1.2 (Ronquist & Huelsenbeck, 2003). Two simultaneous runs (four Markov chains Monte Carlo chains) were conducted for 1.0 × 10^6^ generations with independent models, and every 1,000 generations were sampled. Stationarity was considered to have been reached when the average standard deviation of the split frequencies was below 0.01. The first 25% of the sampled trees and the estimated parameters were discarded as burn-in. The remaining trees were used to calculate the consensus tree and Bayesian posterior probabilities. ML analysis was performed using RAxML 8.1.17 (Stamatakis, 2014). Branch support was assessed using a rapid bootstrapping set to terminate automatically with 10 runs and 1,000 replications using the GTRGAMMA model.

## Results and Discussion

### Mitogenome organisation

We sequenced and annotated mitogenome of the five species (*C. tenuirostris*, *C. alpine*, *C. alba*, *C. subminuta*, and *L. falcinellus*) and obtained four complete and one nearly complete mitogenome. The sequences of the PCR products showed the same results as the next-generation sequences. The circular mitogenome encodes a control region and a typical set of 37 genes containing 2 rRNA genes (12S and 16S rRNA), 13 protein-coding genes (PCGs), and 22 tRNA genes. Among the 37 genes, 9 genes (tRNA^Gln^, tRNA^Ala^, tRNA^Asn^, tRNA^Cys^, tRNA^Tyr^, tRNA^Ser^, ND6, tRNA^Pro^, and tRNA^Glu^) were encoded on the light strand, and the remaining 28 genes were encoded on the heavy strand. The gene numbers, composition, and order are always highly conserved, without any structural rearrangement (Chen et al., 2019; Hu et al., 2017).

Mitogenomes from the genus *Calidris* displayed moderate size variation, with a mean length of 16,747 bp (SD = 87, *n* = 5), ranging from 16,642 bp (*C. alba*) to 16,791 bp (*C. alpina*) (Table 2). The length variation was minimal in PCGs, tRNAs, and rRNA; however, most of the size variation was primarily due to mutations in the control region. Gene overlaps were found at nine gene junctions, spanning 1–10 nucleotides, for a total of 34 bp in length. The longest overlap (10 bp) was located between ATP8 and ATP6, and the COI/tRNA^Ser^ pairs overlapped with nine nucleotides. Intergenic spacer regions were observed 17 times.

### Nucleotide composition

Base composition and strand asymmetry were calculated (Table 2). The overall mean base compositions were A = 31.40%, C = 30.08%, T = 24.84%, and G = 13.64%. The nucleotide composition was consistently AT-biased, ranging from 55.74% (*C. tenuirostris*) to 56.61% (*C. ruficollis*) (Table 2), which is consistent with previous studies (Chen et al., 2019; Hu et al., 2021).

The values of the AT and GC skews are a measure of compositional asymmetry. The mean AT skew value observed was 0.12 ± 0.01 (mean ± SD), ranging from 0.11 to 0.13. The mean GC skew value was –0.37 ± 0.01, ranging from –0.39 to –0.36 (Table 2). A positive value of AT skew and negative value of GC skew was found, which suggested a specific bias toward A and C in nucleotide composition. The mitogenome of *Calidris* had a nucleotide composition and organisation similar to those of *L. falcinellus* and *E. pygmeus*.

### The usage of start and stop codon

**Figure 1:**
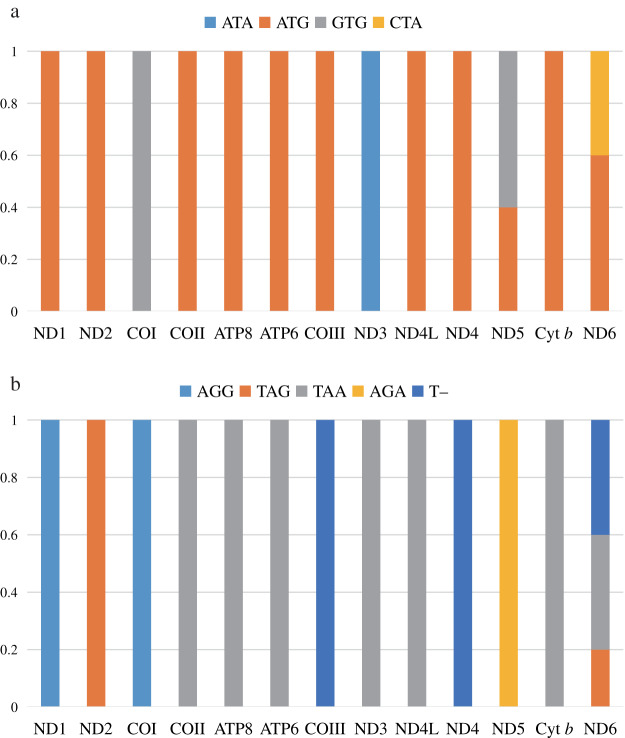
The usage of start codons (A) and stop codons (B) in the 13 protein-coding genes of the five species in this study. All genes are shown in the order of occurrence in the mitochondrial genome starting from ND1.

Four start codons (ATG, GTG, CTA, and ATA) were detected in protein-coding genes (Fig. 1). The most common start codon was ATG, accounting for 76.92% of all start codons, followed by GTG (12.31%), and ATA (7.69%). We found that ATG appeared in 11 protein-coding genes (except for COI and ND3), and 9 genes used onlyATG as the start codon. The start codon GTG was commonly used in COI and ND5, ATA was only used in ND3, but it was frequently observed in other species, and CTA was only used in ND6 (Chakraborty et al., 2022; Uddin, Choudhury & Chakraborty, 2018).

Four stop codons (TAA, TAG, AGG, and AGA) and one incomplete stop codon (T–) were detected (Fig. 1). ND6 had three stop codons (TAA, TAG, and T–), while the others had only one stop codon. The most common stop codon was TAA, which accounted for 49.23% of the stop codons, and appeared in seven protein-coding genes. Subsequently, stop codon T– (18.46%) was used in COIII, ND4, and ND6. This is a common phenomenon in Scolopacidae, which may be completed by poly-adenylation of the mRNA post-transcriptionally (Anderson et al., 1981; Lavrov, Boore & Brown, 2002). The stop codon AGG (15.38%) was used for ND1 and COI. Finally, the stop codon AGA was only found in ND5, commonly found in Scolopacidae and Laridae (Hu et al., 2017; Yoon, Cho & Park, 2015).

**Figure 2:**
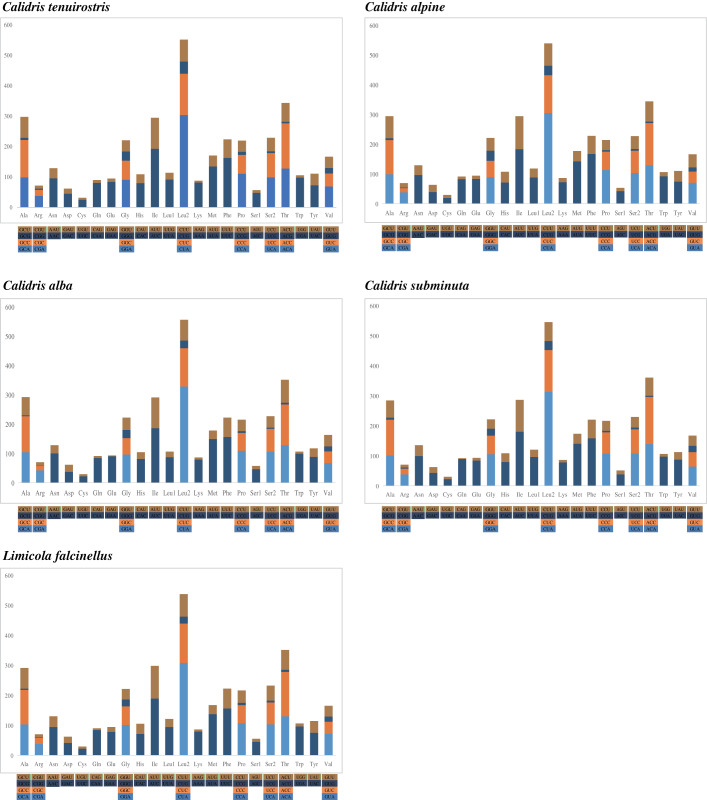
The codon number of the mitogenomes of species in this study, the stop codon is not included.

Nucleotide composition bias was reflected in codon usage patterns. The highest proportion of amino acids was Leu2 (14.2–14.7%), followed by Thr (9.1–9.5%), Ile (7.6–7.9%), Ala (7.5–7.9%), and Ser (7.4–7.7%). Cys was the lowest at <1%. Among the 62 amino acid encoding codons, Leu^CUA^, Ile^AUC^, and Phe^UUC^ were the most frequently used (Fig. 2).

### Variation and evolutionary rates of protein-coding genes

The total length of the protein-coding genes was 11,397 bp after removing termination codons and indels. The length of the 13 protein-coding genes revealed that the ND5 (1,815 bp) and ATP8 (168 bp) genes were the longest and shortest. Comparing each protein-coding gene provides a better understanding of the evolutionary patterns under different selective pressures. The number of variable positions in each gene varied from 15.82% (ND4L) to 25.60% (ATP8), and parsimony-informative sites ranged from 2.36% (ND4L) to 8.93% (ATP8), indicating that the ATP8 contains more variable sites than ND4L. The number of singletons was the lowest in COIII (10.71%) and the highest in ATP8 (16.67%). The average uncorrected pairwise distances (Aupd) revealed that the evolutionary rate for COIII (0.09) and ND4L (0.09), was slow, whereas ND6 (0.13) and ATP8 (0.15) were fast (Table 3). Therefore, we can infer that ATP8 has afast evolutionary rate, while COIII is the most conserved protein-coding gene.

**Table 3:** The mutational information and average distances calculated by 13 protein-coding genes.

Gene	Length (bp)	%Vs	%Pis	%S	%Aupd
ND1	978	22.70	7.46	15.24	12.30
ND2	1,041	21.71	8.17	13.54	11.9
COI	1,551	18.83	7.35	11.48	10.29
COII	684	19.88	8.04	11.84	11.15
ATP8	168	25.60	8.93	16.67	14.68
ATP6	684	21.35	8.19	13.16	11.72
COIII	784	16.84	6.12	10.71	9.05
ND3	352	21.59	7.95	13.64	11.73
ND4L	297	15.82	2.36	13.47	9.16
ND4	1,378	22.86	7.62	15.24	12.76
ND5	1,815	20.06	6.50	13.55	10.77
Cyt *b*	1,151	19.51	6.82	12.69	10.65
ND6	522	21.65	8.62	13.03	13.30

**Note:**

Vs, variable sites; Pis, parsimony informative sites; S, singleton; Aupd, The average uncorrected pairwise distances.

**Figure 3:**
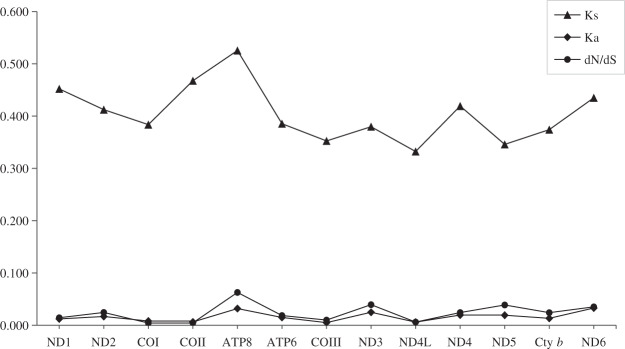
Evolutionary rates of 13 protein-coding genes in five species. Synonymous nucleotide substitutions per synonymous site (Ks) and nonsynonymous nucleotide substitutions per nonsynonymous site (Ka) are calculated using Dnasp, and dN/dS is calculated using DataMonkey.

To better understand the evolutionary patterns of the 13 protein-coding genes and the role of selection, the values of Ka, Ks, and dN/dS (ω) were calculated for each protein-coding gene (Fig. 3). The average Ka value was 0.019, ranging from 0.005 (COIII) to 0.033 (ND6). The average Ks value was 0.471, ranging from 0.332 (ND4L) to 0.526 (ATP8). The highest value of dN/dS was observed in the gene of ATP8 (0.063). The dN/dS values for all protein-coding genes were far lower than one, indicating that these genes evolved under purifying selection.

### Genetic distance

Genetic distance measures the genetic divergence between species or populations (Fregin et al., 2012). In this study, the largest genetic distance was between *E. pygmeus* and *L. falcinellus* (22.5%), and the smallest was between *E. pygmeus* and *C. ruficollis* (12.8%). The genetic distance within the genus *Calidris* varied from 16.0–21.5%. The genetic distance between *Calidris* and *Eurynorhynchus* was 18.2% (Table 4), and that between *Calidris* and *Limicola* was 21.2%. The genetic distance between the three genera was smaller than that between inter-genus *Calidris*.

**Table 4:** The genetic distances between species in genus *Calidris*, *Limicola* and *Eurynorhynchus*, the numbers 1–7 represents *C. tenuirostris*, *C. alpina*, *C. alba*, *C. subminuta*, *C. rubficollis*, *L. falcinellus* and *E*. *pygmeus* respectively.

Species	Genetic distances						
	1	2	3	4	5	6	7
*Calidris tenuirostris*		0.215	0.206	0.198	0.213	0.209	0.218
*Calidris alpina*	0.215		0.174	0.190	0.193	0.212	0.199
*Calidris alb*a	0.206	0.174		0.183	0.185	0.208	0.199
*Calidris subminuta*	0.198	0.190	0.183		0.160	0.209	0.165
*Calidris ruficollis*	0.213	0.193	0.185	0.160		0.222	0.128
*Limicola falcinellus*	0.209	0.212	0.208	0.209	0.222		0.225
*Eurynorhynchus pygmeus*	0.218	0.199	0.199	0.165	0.128	0.225	

### Phylogenetic analysis

**Figure 4:**
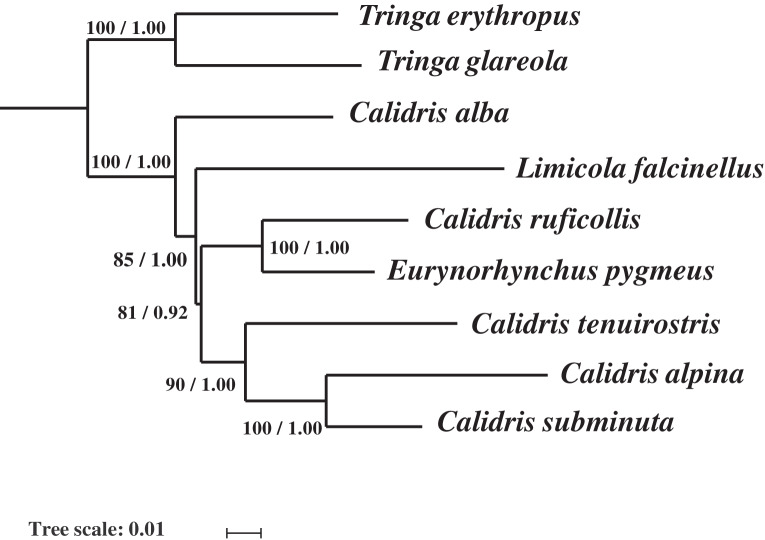
The phylogenetic trees constructed with the 13 protein-coding genes, 12S and 16S rRNA using Bayesian inference and Maximum likelihood. Maximum likelihood bootstrap values and Bayesian percent posterior probabilities are indicated at each node in the tree, separated by ‘/’.

**Figure 5:**
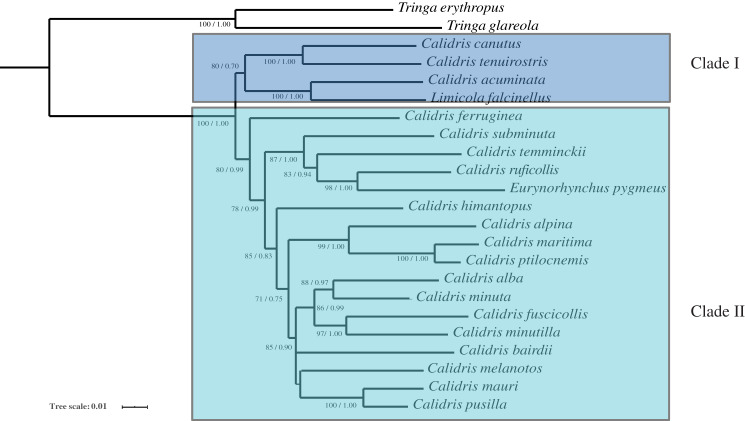
The phylogenetic trees constructed with 12S rRNA, COI and Cyt b using Bayesian inference and Maximum likelihood. Maximum likelihood bootstrap values and Bayesian percent posterior probabilities are indicated at each node in the tree, separated by ‘/’.

Phylogenetic analysis with two inference methods (BI and ML) of 13 mitochondrial protein-coding genes, 12S and 16S (Dataset 1: 13,782 bp in length), for nine species revealed identical topologies, which were highly supported by bootstrap and posterior probabilities at most nodes (Fig. 4). Combined with 12S rRNA, COI, and Cyt *b*, Dataset 2 was 2,238 bp long after alignment. The topologies constructed using Dataset 2 recovered two main clades (clade I and clade II). Clade I contained *L. falcinellus* plus *C. acuminate*, which was a sister group of the to *C. tenuirostris* and *C. canutus* groups (Fig. 5). Within clade II, it is worth noting that *E. pygmeus* was a sister group of *C. ruficollis*. This study was in agreement with previous hypotheses that *Calidris* is not a monophyletic genus, as species from *Eurynorynchus* and *Limicola* were nested within *Calidris* (Gibson & Baker, 2012).

## Conclusions

In this study, we sequenced and annotated the mitogenome of five species (*C. tenuirostris*, *C. alpine*, *C. alba*, *C. subminuta*, and *L. falcinellus*), and obtained four complete and one nearly complete mitogenome. Circular mitogenomes displayed moderate size variation, with a mean length of 16,747 bp (SD = 87, *n* = 5), ranging from 16,642 to 16,791 bp. The mitogenome encoded a control region, and a typical set of 37 genes containing 2 rRNA genes, 13 protein-coding genes and 22 tRNA genes. There were four start codons, four stop codons, and one incomplete stop codon (T–). The nucleotide composition was consistently AT-biased. The average uncorrected pairwise distances revealed heterogeneityin the evolutionary rate for each gene. COIII had a slow evolutionary rate, whereas ATP8 gene had a fast rate. dN/dS analysis indicated that the protein-coding genes were under purifying selection. The genetic distances between species showed that the greatest genetic distance was between *Eurynorhynchus pygmeus* and *Limicola falcinellus* (22.5%), and the shortest was between *E. pygmeus* and *C. ruficollis* (12.8%). The phylogenetic trees based on entire mitogenomes demonstrated that *E. pygmeus* was a sister species to *C. ruficollis*, whereas *L. falcinellus* was more distantly related to the other species within the genus *Calidris*. Our study suggests that *Calidris* is not a monophyletic genus, as species from the genera *Eurynorynchus* and *Limicola* were nested within *Calidris*. The molecular data obtained in this study are valuable for research on the taxonomy, population genetics, and evolution of birds in the genus *Calidris*.

## Supplemental Information

10.7717/peerj.13268/supp-1 Supplemental Information 1 Species used for phylogenetic analysis in this study.

10.7717/peerj.13268/supp-2 Supplemental Information 2 The nucleotide substitution models for mitochondrial data.

## Additional Information and Declarations

### Data Availability

The following information was supplied regarding data availability: The data is available at NCBI GenBank: MW160419, MW168383, MW168384, MW168385, MW160420; BioProject: PRJNA679413; and Bio-Sample: SAMN16833227, SAMN16833228, SAMN16833229, SAMN16833230, and SAMN16833231.
