# Supplementary material for: Five new mitogenomes sequences of Calidridine sandpipers (Aves: Charadriiformes) and comparative mitogenomics of genus Calidris
Source: PeerJ. 2022 Apr 18;10:e13268. doi: 10.7717/peerj.13268 (PMC9022639; doi:10.7717/peerj.13268)
Supplement: Supplemental Information 1 [file peerj-10-13268-s001.docx]

Table S1 Species used for phylogenetic analysis in this study.

| Order | English name | Species | Mitogenome | COI | Cyt *b* | 12S |
| --- | --- | --- | --- | --- | --- | --- |
| 1 | Red-necked Stint | *Calidris ruficollis* | MG736926 |  |  |  |
| 2 | Great Knot | *Calidris tenuirostris* | MW160419 |  |  |  |
| 3 | Dunlin | *Calidris alpina* | MW168383 |  |  |  |
| 4 | Sanderling | *Calidris alba* | MW168384 |  |  |  |
| 5 | Long-toed Stint | *Calidris subminuta* | MW168385 |  |  |  |
| 6 | Western Sandpiper | *Calidris mauri* |  | AY666261 | KC969161 | KF041190 |
| 7 | Red-necked Stint | *Calidris pusilla* |  | AY666222 | KC969166 | KF041195 |
| 8 | Pectoral Sandpiper | *Calidris melanotos* |  | AY666286 | KC969162 | KF041191 |
| 9 | White-rumped Sandpiper | *Calidris fuscicollis* |  | AY666305 | KC969158 | KF041187 |
| 10 | Least Sandpiper | *Calidris minutilla* |  | AY666246 | KC969164 | KF041193 |
| 11 | Little Stint | *Calidris minuta* |  | GQ481439 | KC969163 | KF041192 |
| 12 | Baird's Sandpiper | *Calidris bairdii* |  | AY666373 | KC969155 | KF041184 |
| 13 | Purple sandpiper | *Calidris maritima* |  | KF009524 | KC969160 | KF041189 |
| 14 | Rock Sandpiper | *Calidris ptilocnemis* |  | DQ433414 | AY156262 | KF041194 |
| 15 | Temminck's Stint | *Calidris temminckii* |  | GU571784 | KC969169 | KF041198 |
| 16 | Curlew Sandpiper | *Calidris ferruginea* |  | GQ481438 | KC969157 | KF041186 |
| 17 | Stilt Sandpiper | *Calidris himantopus* |  | AY666359 | KC969159 | KF041188 |
| 18 | Sharp-tailed Sandpiper | *Calidris acuminata* |  | MK261984 | KC969152 | KF041181 |
| 19 | Red Knot | *Calidris canutus* |  | AY666343 | KC969156 | KF041185 |
| 20 | Broad-billed Sandpiper | *Limicola falcinellus* | MW160420 |  |  |  |
| 21 | Spoon-billed Sandpiper | *Eurynorhynchus pygmeus* | KP742478 |  |  |  |
| 22 | Northern Lapwing | *Vanellus vanellus* | KM577158 |  |  |  |
| 23 | Wood Sandpiper | *Tringa glareola* | KY128485 |  |  |  |
