# Supplementary material for: Five new mitogenomes sequences of Calidridine sandpipers (Aves: Charadriiformes) and comparative mitogenomics of genus Calidris
Source: PeerJ. 2022 Apr 18;10:e13268. doi: 10.7717/peerj.13268 (PMC9022639; doi:10.7717/peerj.13268)
Supplement: Supplemental Information 2 [file peerj-10-13268-s002.docx]

Table S2 The nucleotide substitution models selected for mitochondrial data.

| **Subset** | **Gene** | **Length (bp)** | **Best Model** |
| --- | --- | --- | --- |
| **Dataset1** |  |  |  |
|  | 12S | 979 | TIM3+F+R2 |
|  | 16S | 1608 | TN+F+I |
|  | ND1 | 978 | TPM2+F+I |
|  | ND2 | 1041 | K3Pu+F+G4 |
|  | COI | 1551 | TIM2+F+G4 |
|  | COII | 684 | TIM2+F+G4 |
|  | ATP8 | 168 | TN+F+I |
|  | ND4 | 1378 | TIM2+F+R3 |
|  | ATP6 | 684 | TIM2+F+R3 |
|  | COIII | 784 | TIM2+F+I+G4 |
|  | Cyt *b* | 1143 | TIM2+F+I+G4 |
|  | ND3 | 351 | TPM2+F+G4 |
|  | ND4L | 297 | TPM2+F+I |
|  | ND5 | 1816 | TPM2+F+I |
|  | ND6 | 522 | TIM3+F+I+G4 |
| **Dataset2** |  |  |  |
|  | 12S rRNA, | 617 | TIM2+F+I+G4 |
|  | COⅠ | 621 | TIM2+F+R3 |
|  | Cyt *b* | 1005 | TIM2+F+I+G4 |
